# Effect of problem-based learning tutor seniority on medical students’ emotions: an equivalence study

**DOI:** 10.1186/s12909-023-04416-9

**Published:** 2023-06-07

**Authors:** Osamu Nomura, Tatsuki Abe, Yuki Soma, Hirofumi Tomita, Hiroshi Kijima

**Affiliations:** 1 Department of Health Sciences Education, Hirosaki University, Hirosaki, Japan; 2 Centre for Community-Based Health Professions Education, Hirosaki University, Hirosaki, Japan; 3 Department of International Cooperation for Medical Education, International Research Center for Medical Education, Graduate School of Medicine, The University of Tokyo, Tokyo, Japan; 4 Faculty of Education, Hirosaki University, Hirosaki, Japan; 5 Department of Cardiology, Hirosaki University, Hirosaki, Japan

**Keywords:** Near-peer learning, Emotions, Emotional outcomes, Control-Value Theory, Medical education, Problem-based learning, Medical emotion scale

## Abstract

**Background:**

The effectiveness of peer learning has been recognized and discussed by many scholars, and implemented in the formal curriculums of medical schools internationally. However, there is a general dearth of studies in measuring the objective outcomes in learning.

**Methods:**

We investigated the objective effect of near-peer learning on tutee’s emotions and its equivalence within the formal curriculum of a clinical reasoning Problem Based Learning session in a Japanese medical school. Fourth-year medical students were assigned to the group tutored by 6^th^-year students or by faculties. The positive activating emotion, positive deactivating emotion, negative activating emotion, negative deactivating emotion, Neutral emotion were measured using the Japanese version of the Medical Emotion Scale (J-MES), and self-efficacy scores were also assessed. We calculated the mean differences of these variables between the faculty and the peer tutor groups and were statistically analyzed the equivalence of these scores. The equivalence margin was defined as a score of 0.4 for J-MES and 10.0 for the self-efficacy score, respectively.

**Results:**

Of the 143 eligible participant students, 90 were allocated to the peer tutor group and 53 were allocated to the faculty group. There was no significant difference between the groups. The 95% confidence interval of the mean score difference for positive activating emotions (–0.22 to 0.15), positive deactivating emotions (–0.35 to 0.18), negative activating emotions (–0.20 to 0.22), negative deactivating emotions (–0.20 to 0.23), and self-efficacy (–6.83 to 5.04) were withing the predetermined equivalence margins for emotion scores, meaning that equivalence was confirmed for these variables.

**Conclusions:**

Emotional outcomes were equivalent between near-peer PBL sessions and faculty-led sessions. This comparative measurement of the emotional outcomes in near-peer learning contributes to understanding PBL in the field of medical education.

## Background

Peer learning is defined as “learning with and from each other” [1]. It involves a network of learning relationships among multiple learners where ideas, knowledge, and experiences are shared for mutual benefit. A common form of peer learning in health professions education is near-peer learning, where senior students teach junior students. The effectiveness of senior students as peer tutors to teach junior students in undergraduate medical education have been widely studied, with results suggesting that in selected contexts, short-term learner outcomes are comparable with those produced by faculty-based learning [2].

Previous systematic reviews have shown that near-peer leaning in medical education leads to improvements in knowledge and clinical skills compared with traditional faculty-led learning [3–6]. Scholars have theorized why near-peer learning is beneficial to learners in medical education. Some of the more prominent of those concepts include the presence of “cognitive congruence” between student learners (tutee) and student teachers (tutors), as similarity in the knowledge basis that is shared between the tutee and tutors; and “social congruence” which refers to tutees and peer tutors in similar social roles sharing an informal style of communication, fostering a more emotionally supportive atmosphere where exchange of ideas is facilitated [7, 8].

Thus, the benefit of peer learning is discussed conceptually as the emotional state of the tutee, such as increased feelings of relaxation and reduced stress; however, few studies have examined the emotional outcomes of tutees taught by peer tutors in the context of medical education. In addition, previous studies that have suggested emotional benefits of peer learning in tutees have mostly reported qualitative results rather than objective quantitative evidence [9, 10]. Therefore, there is a need to investigate the effects of near-peer learning on objective emotional outcomes compared to faculty-led teaching.

Pekrun’s Control-Value Theory of Achievement Emotions postulates that learners’ emotions will influence the degree to which they learn to do a given task and are a widely used as the theoretical framework in medical education research [11, 12]. The Medical Emotion Scale was developed to assess medical trainees’ achievement emotions by applying this theory to relevant medical education contexts [13]. We have previously developed a Japanese version of the Medical Emotion Scale (J-MES) by translating the MES into Japanese and evaluating validity evidence [14, 15].

### Study aim

The aim of the study was to investigate the effect of near-peer learning on tutee’s achievement emotions, and its equivalence in an objective manner using the J-MES.

## Methods

### Program structure

The study was conducted within the structure of one Problem Based Learning “Cycle” of clinical reasoning within the formal curriculum of the PBL 4^th^-year course at Hirosaki University in Japan, in November 2021. Each Cycle of PBL consists of tasks presented in the following five sessions: Session 1: group discussion for problem identification within a case scenario; Sessions 2 and 3: self-study sessions; Session 4: group discussion where the final diagnosis is shown; and Session 5: concluding lecture. These five sessions are usually delivered in one week. There are 7–9 students in each PBL group, and each group has one tutor. Our PBL has implemented the near-peer learning for three years, and the tutor was either a 6^th^-year medical student or a faculty member.

#### Participants

We invited 150 4^th^-year students to participate in the study, of which 146 agreed to participate. The students were divided into a near-peer tutored group and a faculty tutored group without systematic randomization (i.e., in alphabetical order of the students’ last names).

#### Tutor selection and tutor training

All 6^th^-year students experienced two training sessions in the first half of the semester (weeks 1–6), including mandatory viewing of a one-hour tutor-training video before the session. The instructors participating in PBL were faculty members (i.e., professor, associate professor, instructor, assistant professor, and teaching and research associate) assigned from all departments of our medical school. Faculty tutors were also required to watch a one-hour tutor training video before the PBL session.

#### Measurements

The participants completed an online survey immediately after Session 1 of one PBL “Cycle” during the study period. The survey was presented via Survey Monkey and included items regarding the student demographics, J-MES, the perceived level of difficulty of the PBL scenario case, and post-task self-efficacy for clinical reasoning.

**Fig. 1:**
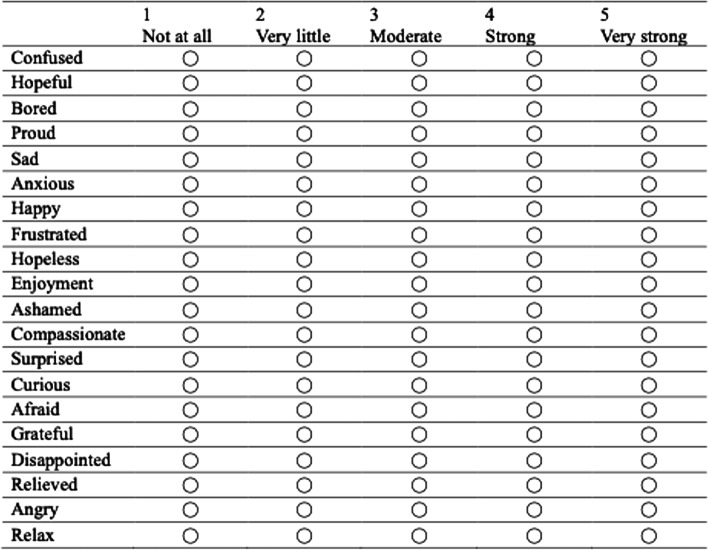
Items of the Medical Emotion Scale

**Fig. 2:**
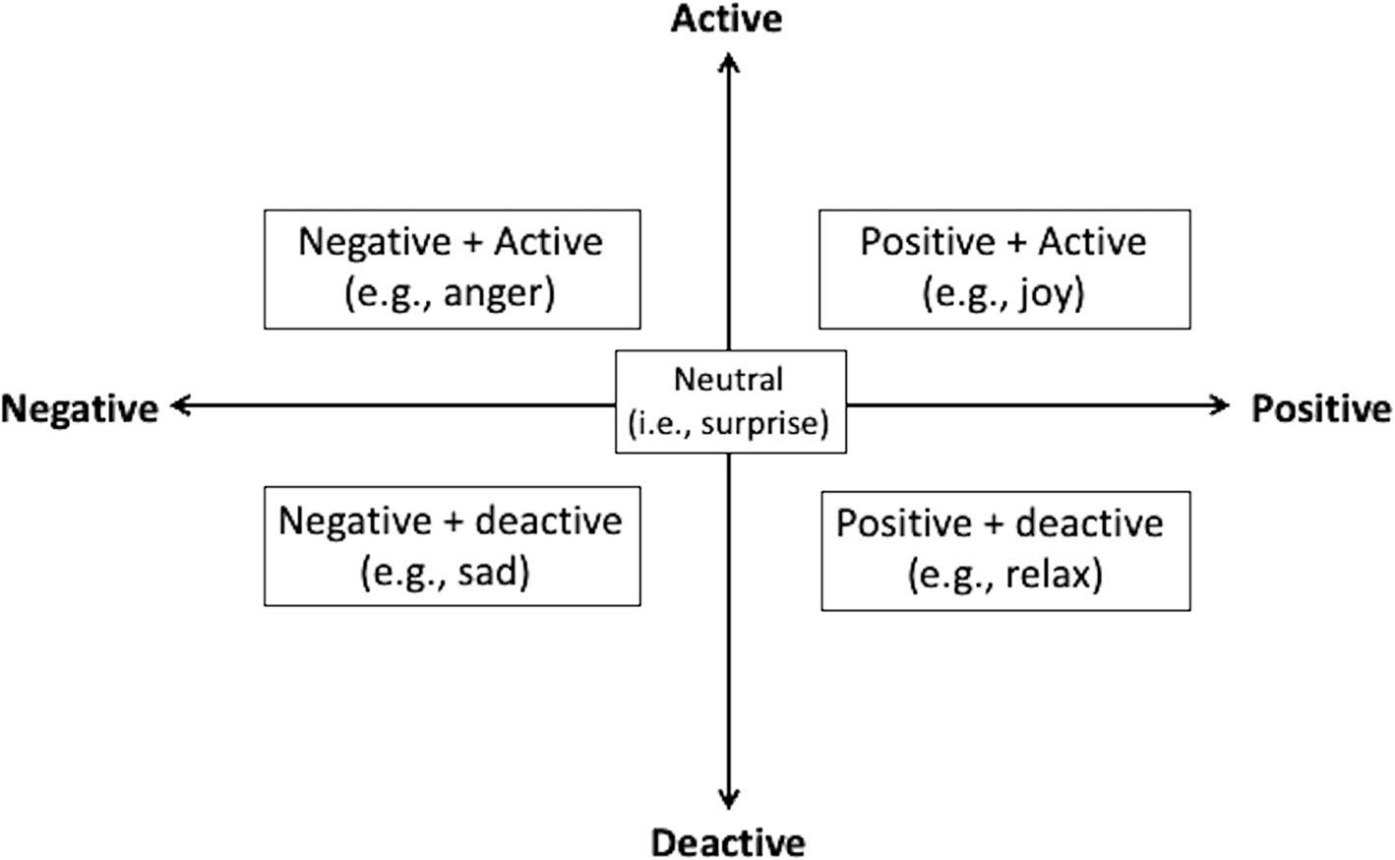
Category of the Emotion Items

The J-MES consists of 20 items containing adjectives that describe discrete emotions using a five-point Likert scale (Fig. 1) [13, 14]. The items are categorized into five subscales (Fig. 2) according to the valence (positive/negative) and activation (active/deactive) of the emotions: (a) positively activating (e.g., happiness, hope); (b) positively deactivating (e.g., relaxed, relieved); (c) negatively activating (e.g., anger, shame), (d) negatively deactivating (e.g., sadness, boredom), and neutral (i.e., surprise). The perceived level of difficulty of the PBL scenario was assessed using a 10-point Likert scale, and post-task self-efficacy for clinical reasoning was assessed using a 100-point Likert scale with 10-point intervals [14, 16].

#### Equivalence study design

We employed an equivalence design to test our hypothesis that the educational outcomes of near-peer learning would be equivalent to those of faculty-led instruction. This study design has been widely used in clinical trials, for example, to demonstrate the clinical effectiveness of a new cost-effective treatment by showing that patients receiving the new treatment have equivalent outcomes to patients receiving the standard treatment [17]. The concept of equivalence has recently been applied in the context of medical education [18]. The financial, material, and human resources for the education of medical trainees are increasing in this area as teaching strategies have become more diverse than before; therefore, faculties are looking for new cost-effective strategies that provide the same results as the standard ones [19]. In terms of near-peer learning for PBL, it is particularly valuable for institutions to use methods that are less costly in terms of faculty human resources and to promote the development of senior students’ teaching skills by having them act as PBL tutors. We defined the equivalence range as a score of 0.4 for J-MES. A score of 10.0 for self-efficacy was considered pedagogically meaningful, as 10% of scores are conventionally set as the margin.

#### Statistical analysis

We calculated the mean and standard deviation (SD) for continuous variables and proportions for categorical variables. The unpaired t-test, χ^2^ test, and Fisher’s exact test were used to compare variables between the tutor groups. The mean differences of positive activating emotion, positive deactivating emotion, negative activating emotion, negative deactivating emotion, neutral emotion, and self-efficacy scores between the faculty and the peer tutor groups were calculated. Equivalence between the tutor groups was examined by two one-sided test procedures and the 95% confidence interval was calculated for mean score difference using R with the “equivalence” package (version 0.7.2). All statistical analyses were performed using R software and the significance level was set at *p* < 0.05.

## Results

**Fig. 3:**
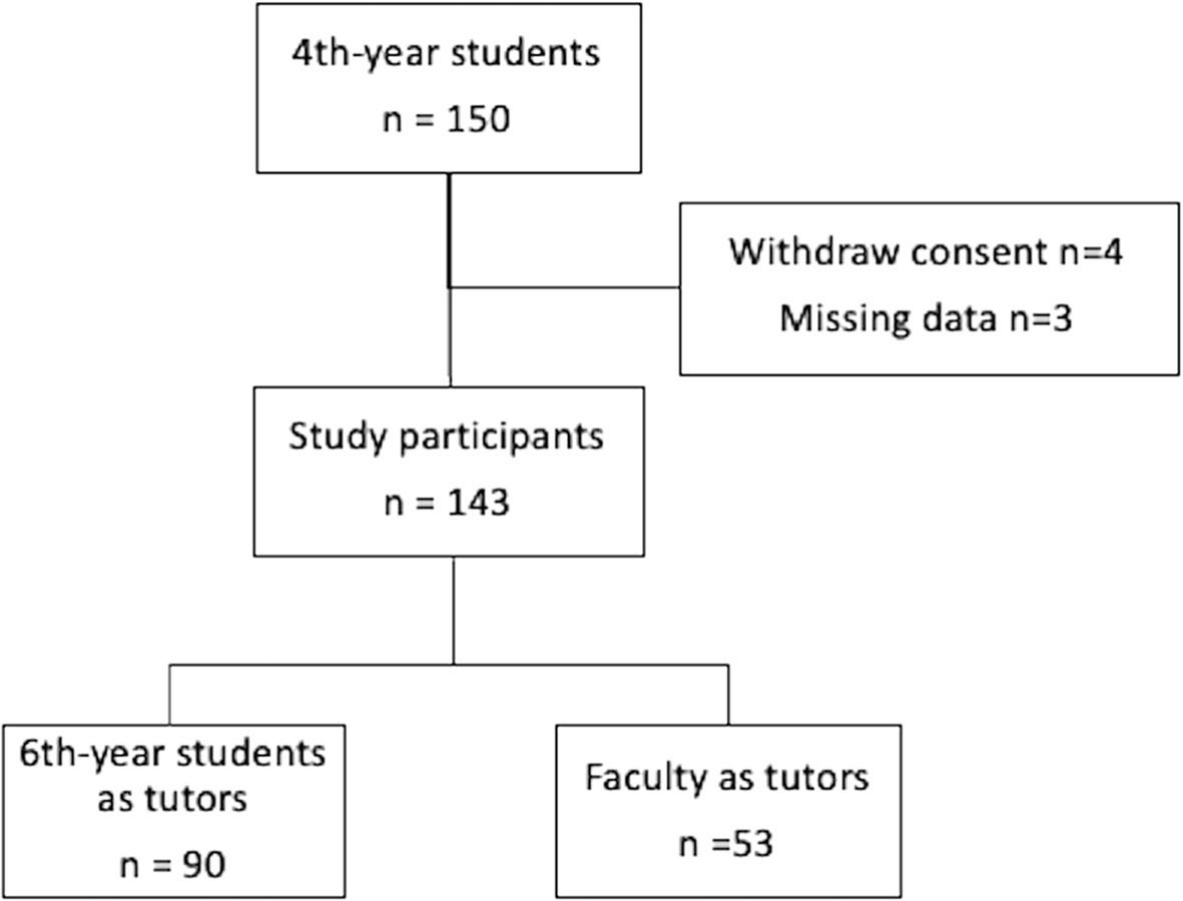
Study Flow

Of the 150 recruited 4^th^-year students invited to participate, 4 students withdrew consent and 3 students had missing data. Among the 143 remaining participants, 90 were allocated with 6^th^-year students as tutors, and 53 with faculty as tutors (Fig. 3).

### Participant characteristics

There were 53 students in the faculty tutored group and 90 students in the near-peer tutored group. There were no statistical differences between these groups in terms of male sex (54.7% vs. 48.9%, *p* = 0.501), proportion of graduate entry student (11.3% vs. 16.7%, *p* = 0.383), proportion of regional quota system students (49.1% vs. 47.8%, *p* = 0.882), mean age of students (23.4 vs. 24.0, *p* = 0.354), and mean score of perceived difficulty (7.5 vs. 7.2, *p* = 0.490) (Table 1).

**Table 1:** Attributes of participants

	Faculty (*n* = 53)	Peer tutor (*n* = 90)	*p* value
Male, n (%)	29 (54.7)	44 (48.9)	0.501
Bachelor, n (%)	6 (11.3)	15 (16.7)	0.383
Regional quota system students, n (%)	26 (49.1)	43 (47.8)	0.882
Age, mean (SD) (years)	23.4 (3.3)	24.0 (4.9)	0.354
Perceived difficulty, mean (SD)	7.5 (1.8)	7.2 (2.0)	0.490

### Emotion score comparison between groups

In assessment of emotion score using the J-MES, the 95% confidence interval of the mean score difference for positive activating emotions, positive deactivating emotions, negative activating emotions, negative deactivating emotions, and self-efficacy were within the predetermined equivalence margins for emotion scores (Table 2), indicating that equivalence was confirmed regarding these variables.

**Table 2:** Equivalence analysis using emotion scores and self-efficacy

	Faculty (*n* = 53)	Peer tutor (*n* = 90)	Mean difference	95% confidence interval	Equivalence margin
Positive activating emotions, mean (SD)	3.04 (0.69)	3.01 (0.62)	–0.03	–0.22 to 0.15	–0.4 to 0.4
Positive deactivating emotions, mean (SD)	3.02 (0.98)	2.93 (0.90)	–0.09	–0.35 to 0.18	–0.4 to 0.4
Negative activating emotions, mean (SD)	2.11 (0.81)	2.12 (0.60)	0.01	–0.20 to 0.22	–0.4 to 0.4
Negative deactivating emotions, mean (SD)	1.79 (0.78)	1.81 (0.73)	0.01	–0.20 to 0.23	–0.4 to 0.4
Neutral emotion, mean (SD)	2.62 (1.06)	2.63 (1.12)	0.01	–0.30 to 0.32	–0.4 to 0.4
Self-efficacy, mean (SD)	51.77 (20.06)	50.88 (21.06)	–0.90	–6.83 to 5.04	–10.0 to 10.0

## Discussion

To the best of our knowledge, this is the first study to compare the emotional outcomes of near-peer PBL tutoring and faculty-led PBL. We were able to demonstrate equivalence of peer tutors against faculty for PBL tutoring in terms of perceived emotions and self-efficacy of the tutees. The role of students’ perceived emotions is deemed significant in education, supported by increasing evidence from decades of cognitive science research that it has an indispensable catalytic role in learning. Emotion modulates many cognitive processes, including perception, memory, attention, cognitive reasoning and psychomotor skills [20, 21]. Thus, emotions could be a surrogate representation of performance of learning.

Our finding is consistent with previous reports that near-peer PBL seems to have a positive impact on learner satisfaction, and academic performance does not suffer relative to faculty tutoring [22, 23]. This is not to say that it does not matter who the tutor is, but rather that near-peers compensate for the relative lack in content knowledge and display a high cognitive and social congruence towards students. The relaxed atmosphere or learning climate fostered by the senior students is thought to facilitate learning [24] and is deemed an important factor for development in faculty programs [25]. Experienced faculty members can create positive learning climates in which students can learn without feeling pressured; however, peers and near-peers are in better position to do this as they are usually perceived as less threatening by students and often have a rich understanding of the situation of the curriculum as well as existing knowledge [26]. As such, it can be said that the students’ perception of the learning environment and the emotions that are evoked in the learning atmosphere are important components of the learning experience.

The benefits of using peer tutors for PBL learning are manifold and its necessity has been discussed extensively by many prominent scholars [3–8]. Considering its impact in terms of reduction in cost of human resource and faculty involvement, as well as provision of training opportunity for the peer tutors, it is understandable that many institutions try to incorporate peer teaching into their formal curriculums, even when their long-term benefits are unconfirmed due to the complexity of objective measurement. However, it is important to note that the economic and faculty involvement aspects have not been measured precisely or well studied [27]. In addition, it is not justifiable to ignore the educational burden of student tutors; therefore, curriculum directors need to carefully monitor senior tutors to ensure that their tutoring responsibilities do not become overwhelming for them. In our program, we assigned each student tutor to only one PBL “cycle,” and they tutored two sessions (Session 1 &4) during the semester to optimize their educational experience and burden.

There are some limitations in this study. First, evaluation was conducted in only one session of one PBL cycle; thus, the emotional state of students was not investigated throughout the entire module or semester. Second, we limited the measurements to emotional and self-efficacy variables to simplify the questionnaire, with the aim of achieving a high response rate. However, the relationship between emotions and other related constructs such as motivational briefs need to be added in future studies. Third, the literature has suggested that the relationship between junior and senior students in the East Asian context may be different from that in Western culture, i.e., the relationship in East Asia is more hierarchical than in the West [28]. Therefore, our findings may not be generalizable to the Western context. Larger longitudinal studies that employ various measurements and contexts are needed to overcome these limitations.

In conclusion, we investigated the effect of near-peer learning on tutee’s emotions and its equivalence to faculty tutoring using the Japanese version of the Medical Emotion Scale (J-MES) in the Japanese context. The findings confirmed equivalent effects of near-peer tutors and faculty tutors on the emotions of students in clinical reasoning PBL.

## Data Availability

The datasets used and/or analyzed during the current study are available from the corresponding author on reasonable request.

## Abbreviations

PBL: Problem Based Learning
J-MES: Japanese version of the Medical Emotion Scale

## References

[1] Ten Cate O, Durning S (2007). Dimensions and psychology of peer teaching in medical education. Med Teach.

[2] Nomura O, Onishi H, Kato H. Medical students can teach communication skills – a mixed methods study of cross-year peer tutoring. BMC Med Educ. 2017;17:103. 10.1186/s12909-017-0939-7 PMC5472895 28619020

[3] Hill A, Yu, Wilson, Hawken, Singh, Lemanu. Medical students-as-teachers: a systematic review of peer-assisted teaching during medical school. Adv Med Educ Pract. 2011;2:157–72. 10.2147/AMEP.S14383 PMC3661256 23745087

[4] Brierley C, Ellis L, Reid ER (2022). Peer-assisted learning in medical education: A systematic review and meta-analysis. Med Educ.

[5] Zhang Y, Maconochie M (2022). A meta-analysis of peer-assisted learning on examination performance in clinical knowledge and skills education. BMC Med Educ.

[6] Tai J, Molloy E, Haines T, Canny B (2016). Same-level peer-assisted learning in medical clinical placements: a narrative systematic review. Med Educ.

[7] Ross MT, Cameron HS. Peer assisted learning: a planning and implementation framework: AMEE Guide no. 30. Med Teach. 2007;29(6):527–45. 10.1080/01421590701665886 17978966

[8] Burgess A, Dornan T, Clarke AJ, Menezes A, Mellis C. Peer tutoring in a medical school: perceptions of tutors and tutees. BMC Med Educ. 2016;16(1). 10.1186/s12909-016-0589-1 PMC4784332 26956642

[9] Moore KA, O’Brien BC, Thomas LR (2020). “I Wish They Had Asked”: a Qualitative Study of Emotional Distress and Peer Support During Internship. J Gen Intern Med.

[10] Alexander SM, Dallaghan GLB, Birch M (2022). What Makes a Near-Peer Learning and Tutoring Program Effective in Undergraduate Medical Education: a Qualitative Analysis. Med Sci Educ.

[11] Pekrun R, Perry RP. “Control-value theory of achievement emotions”. In: Reinhard Pekrun, Linnenbrink-Garcia L. International Handbook of Emotions in Education. Routledge; 2014. p. 130–51.

[12] Bieleke M, Gogol K, Goetz T, Daniels L, Pekrun R. The AEQ-S: A Short Version of the Achievement Emotions Questionnaire. Contemp Educ Psychol. 2021;65:101940.

[13] Duffy MC, Lajoie SP, Pekrun R, Lachapelle K. Emotions in medical education: examining the validity of the Medical Emotion Scale (MES) across authentic medical learning environments. Learn Instruct. 2020;70:101150.

[14] Nomura O, Wiseman J, Sunohara M, Akatsu H, Lajoie SP. Japanese medical learners’ achievement emotions: accounting for culture in translating Western medical educational theories and instruments into an asian context. Adv Health Sci Educ. 2021;26:1255–76. 10.1007/s10459-021-10048-9 PMC8452569 33978878

[15] Nomura O, Sunohara M, Watanabe I, Itoh T (2023). Evaluating Emotional Outcomes of Medical Students in Pediatric Emergency Medicine Telesimulation. Children.

[16] Nomura O, Itoh T, Mori T, Ihara T, Tsuji S, Inoue N, Carrière B (2021). Creating Clinical Reasoning Assessment Tools in Different Languages: Adaptation of the Pediatric Emergency Medicine Script Concordance Test to Japanese. Front Med.

[17] Tolsgaard MG, Kulasegaram KM, Ringsted C (2017). Practical trials in medical education: linking theory, practice and decision making. Med Educ.

[18] Brydges R, Fiume A, Grierson L (2022). Mastery versus invention learning: Impacts on future learning of simulated procedural skills. Adv Health Sci Educ.

[19] Klasen M, Sopka S (2021). Demonstrating equivalence and non-inferiority of medical education concepts. Med Educ.

[20] Vuilleumier P (2005). How brains beware: neural mechanisms of emotional attention. Trends Cogn Sci.

[21] Jung N, Wranke C, Hamburger K, Knauff M. How emotions affect logical reasoning: evidence from experiments with mood-manipulated participants, spider phobics, and people with exam anxiety. Front Psychol. 2014;5(570). Available from: https://www.ncbi.nlm.nih.gov/pmc/articles/PMC4050437/. 10.3389/fpsyg.2014.00570 PMC4050437 24959160

[22] Ten Cate O, Durning S (2007). Peer teaching in medical education: twelve reasons to move from theory to practice. Med Teach.

[23] Slavin RE (1990). Research on cooperative learning: consensus and controversy. Educ Leadersh.

[24] Bulte C, Betts A, Garner K, Durning S (2007). Student teaching: views of student near-peer teachers and learners. Med Teach.

[25] Johansson J, Skeff K, Stratos G (2009). Clinical teaching improvement: The transportability of the Stanford Faculty Development Program. Med Teach.

[26] Lockspeiser TM, O’Sullivan P, Teherani A, Muller J (2006). Understanding the experience of being taught by peers: the value of social and cognitive congruence. Adv Health Sci Educ.

[27] Williams B, Reddy P (2016). Does peer-assisted learning improve academic performance? A scoping review. Nurse Educ Today.

[28] Machi, S. Transcending the senpai ‘senior’/kohai ‘junior’boundary through cross-speaker repetition in Japanese. Pragmatics. 2023. 10.1075/prag.21063.mac.

